# Mortality of Whites and Blacks in the U. S. Army

**Published:** 1882-12-20

**Authors:** 


### *MORTALITY OF WHITES AND BLACKS IN THE U. S. ARMY.*

In the report of Surgeon-General Crane, for the year ending June 30th, 1882, we find that the whole number of white troops are 20,778, and colored, 2,265. The deaths from all causes reported among the whites were 10 per 1,000 of mean strength.

Among the colored, the deaths were 11 per 1,000 of mean strength. There were reported also 245 Indian scouts connected with the army, among whom the mortality was 32 in the 1,000.

The report contains other interesting information, and is gotten up with ability and in good taste.

